# The Roles of P2Y_2_ Purinergic Receptors in Osteoblasts and Mechanotransduction

**DOI:** 10.1371/journal.pone.0108417

**Published:** 2014-09-30

**Authors:** Yanghui Xing, Yan Gu, James J. Bresnahan, Emmanuel M. Paul, Henry J. Donahue, Jun You

**Affiliations:** Division of Musculoskeletal Sciences, Department of Orthopaedics and Rehabilitation, The Pennsylvania State University College of Medicine, Hershey, Pennsylvania, United States of America; University of California Davis, United States of America

## Abstract

We previously demonstrated, using osteoblastic MC3T3-E1 cells, that P2Y_2_ purinergic receptors are involved in osteoblast mechanotransduction. In this study, our objective was to further investigate, using a knockout mouse model, the roles of P2Y_2_ receptors in bone mechanobiology. We first examined bone structure with micro-CT and measured bone mechanical properties with three point bending experiments in both wild type mice and P2Y_2_ knockout mice. We found that bones from P2Y_2_ knockout mice have significantly decreased bone volume, bone thickness, bone stiffness and bone ultimate breaking force at 17 week old age. In order to elucidate the mechanisms by which P2Y_2_ receptors contribute to bone biology, we examined differentiation and mineralization of bone marrow cells from wild type and P2Y_2_ knockout mice. We found that P2Y_2_ receptor deficiency reduces the differentiation and mineralization of bone marrow cells. Next, we compared the response of primary osteoblasts, from both wild type and P2Y_2_ knockout mice, to ATP and mechanical stimulation (oscillatory fluid flow), and found that osteoblasts from wild type mice have a stronger response, in terms of ERK1/2 phosphorylation, to both ATP and fluid flow, relative to P2Y_2_ knockout mice. However, we did not detect any difference in ATP release in response to fluid flow between wild type and P2Y_2_ knock out osteoblasts. Our findings suggest that P2Y_2_ receptors play important roles in bone marrow cell differentiation and mineralization as well as in bone cell mechanotransduction, leading to an osteopenic phenotype in P2Y_2_ knockout mice.

## Introduction

It is well known that mechanical loading of bone results in a variety of biophysical signals that affect bone cell metabolism and differentiation [1]–[3]. Oscillatory fluid flow, one of these biophysical signals, has been demonstrated to be a potent stimulator to osteoblast activity, differentiation, extracellular matrix protein production, and gene expression [4]–[8]. Previously, we reported that oscillatory fluid flow induced MC3T3-E1 osteoblastic cell intracellular calcium mobilization via the inositol 1, 4, 5-trisphosphate pathway and increased steady state mRNA levels of osteopontin, a bone extracellular matrix protein [4]. Others have demonstrated that oscillatory fluid flow can enhance COX-2 protein levels, activate ERK1/2 phosphorylation pathways and cause the release of ATP into extracellular space [9]–[11]. However, the mechanisms by which oscillatory fluid flow initiates osteoblast mechanotransduction pathways are still unclear. Previous studies suggest that various membrane bound structures, such as channels, integrins, and primary cilium, are involved [12]–[14]. Additionally, our studies suggested that the G-protein coupled P2Y_2_ purinergic receptors may also play an important role in osteoblast mechanotransduction [5].

Accumulating evidence suggests mechanical signals induce the release of nucleotides, such as ATP, from bone cells, which then activate nucleotide P2 purinergic receptors in adjacent cells to initiate cellular biological responses [15]–[17]. For example, fluid flow induces ATP release in vascular endothelial cells and smooth muscle cells, and ATP acts on P2 receptors to initiate cellular responses which ultimately regulate vascular tone and blood flow [18], [19]. In addition, ATP released by chondrocytes in response to mechanical loading or inflammation contributes to cartilage destructing processes by activating signaling pathways involved in articular pathology, especially in the early stage of arthritic diseases [20]. More importantly, fluid flow induces ATP release in osteoblastic and osteocytic cells [10]. Recently our observations suggest the extracellular nucleotide ATP is involved in fluid flow induced intracellular calcium mobilization in MC3T3-E1 cells in that oscillatory fluid flow failed to increase intracellular calcium concentration in the presence of apyrase, an enzyme that hydrolyses ATP to AMP [4], [5]. This suggests that extracellular nucleotide ATP and its receptors, P2 receptors, may be critical to initiating cellular responses to biophysical signals in osteoblastic cells.

Consistent with the concept that P2 receptors play an important role in bone remodeling, it has been reported that decreased cancellous and cortical bone mass are found in mice lacking the P2X_7_ receptor [21]. However, our previous studies demonstrated that it is P2Y receptors, not P2X receptors, which are responsible for fluid flow induced intracellular calcium mobilization [5]. In addition, Bowler et al. reported that extracellular ATP, acting via the P2Y receptors, can potentiate the response of human osteoblasts to systemic growth and differentiation factors [22]. Moreover, Orriss et al. reported that ATP and UTP at low concentration strongly inhibit in vitro bone formation by osteoblasts via P2Y2 receptors. They also found 9–17% increases in bone mineral content of hindlimbs of P2Y2 deficient mice. However, the roles of P2Y2 receptor in bone mechanotransduction are still unclear.

In addition to P2Y receptor activation by ATP, Bagchi et al. and Yu et al. have reported that P2Y receptors interact with actin-binding proteins (filamin A) and integrins which are widely believed to play an important role in cell mechanotransduction and adhesion, suggesting that mechanical force may activate P2Y receptors through integrins [23], [24]. Furthermore, P2Y_2_ has been reported to play an important role in cell migration [25]. Take together this evidence suggests a pivotal role of P2Y receptors in bone cell mechanotransduction. Thus, we hypothesize that the P2Y_2_ purinergic signaling pathway, activated by ATP release due to mechanical stimulation, is essential to regulation of bone cell response to fluid flow.

In this study, we employed P2Y_2_ knockout (KO) mice models, to elucidate the role of P2Y_2_ in bone. We first examined bone structure and measured bone mechanical properties in both wild type mice (WT) and P2Y_2_ KO mice. Then, we tested the differentiation and mineralization of bone marrow cells. Next, we compared the response of primary osteoblasts from both WT and P2Y_2_ KO mice to ATP stimulation and oscillatory fluid flow. Finally, ATP releases from both WT and P2Y_2_ KO primary osteoblasts in response to oscillatory fluid flow were quantified. Our observations suggest that P2Y_2_ receptors play an important role in bone metabolism.

## Methods

### Wild Type and Knockout Mice

P2Y_2_ KO mice were generated as previously described [23] and were provided by Dr. Beverly H. Koller (University of North Carolina, Chapel Hill, NC). The genetic backgrounds of the male littermate WT and KO mice used in this study were SV129 (Taconic Farms). The full-time veterinary staff at Pennsylvanian State University College of Medicine took care of the experimental mice fed by regular rodent diet with a normal light-dark cycle. The mouse pups were routinely weaned at 21 days of age, and then small tissues (2–3 mm^2^) from mouse ears were collected for genotyping following the genotype procedures provided by Taconic Farms. This study was carried out in strict accordance with the recommendations in the Guide for the Care and Use of Laboratory Animals of the National Institutes of Health. The protocol was approved by the Committee on the Ethics of Animal Experiments of the Pennsylvanian State University College of Medicine (IACUC No.: 42925). Due to different ages of mice in this study, only male mice were used for bone structure phenotype study to eliminate the effects of female hormones on bone.

### Micro-CT analysis of bone structure

Femurs from the right side of 8 week and 17 week old mice were harvested for micro-CT analysis. The diaphyses were scanned starting at the midpoint of the bone and acquiring 76 slices distally using a Scanco vivaCT 40 (Scanco Medical AG) with scan settings of 55 KVp, 145 µA, and 200 ms integration time. Images were reconstructed as a matrix of 2048×2048×76 isotropic voxels measuring 10.5 µm. Images were gaussian filtered (sigma  = 0.8, support  = 1) and a threshold (24% of full scale) was applied to remove the surrounding soft tissue. The periosteal and endosteal boundaries of the cortical bone were segmented using the Scanco semi-automated edge detection algorithm. Periosteal volume, endosteal volume, bone porosity, cortical bone thickness, and BMD were calculated for the diaphysis of each femur using the Scanco Image Processing Library routines.

### Mechanical Testing for Bone Mechanical Properties

Femurs from 8 week and 17 week old mice were stored in PBS at −80°C before being mechanically tested to failure in three-point bending experiments using a Bose Electroforce Load Frame (EnduraTec MN, USA). The flexural support spans were 8 mm while a loading rate of 1 mm/minute was applied. Femurs were consistently oriented so that loading occurred in the medial to lateral direction. All testing was executed with the bones hydrated and at ambient temperature.

### Cell Culture

Mice at age of 6 to 12 weeks were sacrificed and their femurs and tibias removed. Marrow cells were flushed out with a syringe needle system until the bones appeared white. Marrow cells were collected and cultured in cell differentiation medium (α–MEM with 10% FBS, 50 µg/ml L-Ascorbic Acid, 10 nM Dexamethasone, 10 mM Beta-Glycerol-phosphate, 1% P/S) in six well plates. At day 7, 14 and 21, cells were stained for alkaline phosphatase (AP) and extracellular deposited calcium. While bone marrow cells were widely employed as an ideal cell model to examine cell differentiation and mineralization, osteoblasts/osteocytes are major cells in bone to sense mechanical signals and maintain dynamic functions. Thus, primary osteoblastic cells were used to examine bone cell responses induced by mechanical loading. To isolate osteoblastic cells, bones were incubated with 0.01 percent collagenese for 70 minutes and then chopped into small chips with a size approximately 1–2 mm in diameter. Bone chips were then cultured with anti-contamination maintenance medium (α-MEM with 15%FBS, 1% Antibiotic-Antimycotic, 50 µg/ml L-Ascorbic Acid) for 5 days. The medium was switched to normal maintenance media for another 21 days. Cells migrating out of the chips were trypsinized and removed to another larger culture plate for further growth. Finally, cells were subcultured onto glass slides and subjected to oscillatory fluid flow.

### Alkaline phosphatase staining and quantification

For AP staining, a commercially available kit (Sigma) was used. Cells were fixed and stained following manufactures instructions. For quantification, AP activity was determined by the colorimetric conversion of p-nitrophenol phosphate to p-nitrophenol (Sigma) and normalized to total protein (Pierce).

### Calcium Staining and quantification

Extracellular calcium was identified using the von Kossa method. Briefly, cells were fixed with 4% formaldehyde, then incubated with 5% sliver nitrate for 20 minutes, and rinsed with distilled water three times. To quantify calcium, the o-cresolphthalein method was used following instructions from the calcium assay kit (Cayman Chemicals).

### RT–PCR analysis

Cells were lysed and homogenized with a QIAshredder mini column (QIAGEN). Total RNA was extracted with Qiagen RNeasy mini kit. cDNA was prepared from 1 µg total RNA using the iScript Kit (Bio-rad). PCR amplification was performed in a 30 µl reaction with 1 µl cDNA reaction using Qiagen PCR kit according to the manufacturer's protocol. For comparison between the cells from WT and P2Y_2_ KO mice, RT-PCR was performed and the products were analyzed by agarose gel electrophoresis. The primers for mouse osteocalcin were forward 5′-CAG GAG GGC AAT AAG GTA GT-3′ and reverse 5′-GAG GAC AGG GAG GAT CAA G-3′. The primers for mouse β-actin as controls were forward 5′-AGA GGG AAA TCG TGC GTG AC-3′ and reverse 5′-CAA GAA GGA AGG CTG GAA AA-3′.

### Fluid Flow Experiments

To expose cells to oscillatory fluid flow, slides were positioned in parallel plate flow chambers and connected to a servopneumatic materials testing device (EnduraTec), oscillating at 1Hz, via glass Hamilton syringes and rigid wall tubing. Flow rate was monitored in real time with an ultrasonic flowmeter (Transonic Systems). We utilized a flow regime that facilitated the oscillatory movement of a defined volume of fluid across the cell monolayer. Oscillatory fluid flow mimics the shear stresses associated with the loading and unloading of long bones during normal gait and, as such, was implemented at a physiological frequency of 1 Hz (i.e., 1 step/s). For all experiments cells were exposed to fluid flow sufficient to induce 10 dynes/cm^2^ shear stress.

### Western immunoblotting

To examine the effect of fluid flow on ERK1/2 phosphorylation, osteoblasts were exposed to oscillatory fluid flow inducing a shear stress at 10 dynes/cm^2^ for 5 min. Immediately after fluid flow, slides with cells were frozen in −80°C and cells lysed with radio-immunoprecipitation assay buffer (40 mM Hepes (pH 7.4), 1% Triton X-100, 0.5% Na-deoxylcholate, 0.1% SDS, 100 mM NaCl, 1 mM EDTA and 25 mM β-glycerolphosphate) supplemented with 0.2 mM Na_3_VO_4_ and a protease inhibitor cocktail (Calbiochem). The total protein concentrations in cell lysates was quantified with a BCA protein assay kit (Pierce). 15 µg protein from each sample was resolved by SDS-PAGE and transferred to PVDF membranes. The membrane was then probed with a Phospho-ERK1/2 antibody. Total ERK1/2 was used as a control. Visualization of immunoreactive proteins was achieved employing an ECL detection system and membrane exposure to film. Densitometric analysis was carried out with Quality One image analysis software (Bio-Rad).

### ATP release

After oscillatory fluid flow exposure, samples of conditioned media were collected and immediately stored at −80°C until analyzed. ATP concentration in samples of conditioned media was determined using a commercially available ATP bioluminescence kit (Roche). ATP in 50 µl of each sample serves as a co-factor for luciferase, to convert D-luciferin, in 50 µl of a luciferin-luciferase assay buffer, into oxyluciferin and light. The luminescence from each reaction, as measured by a Monolight 3010 luminometer (BD Pharmingen), was compared with a standard curve created by serially diluting an ATP standard. Duplicate measurements were taken from each sample. Control experiments were performed with each pharmacological inhibitor to ensure that they had no detrimental effect on the reaction. Results were normalized to cellular protein concentration using bicinchoninic acid assay (Pierce).

### Data Analysis

The results were analyzed using the statistical package MINITAB (Minitab Inc) and were expressed as the mean ± standard error (SE). To compare observations, the non-parametric Mann-Whitney test was used in which sample variance was not assumed to be equal. p<0.05 was considered statistically significant.

## Results

### Micro-CT Analysis of WT and P2Y_2_ KO mice bone structure

**Figure 1:**
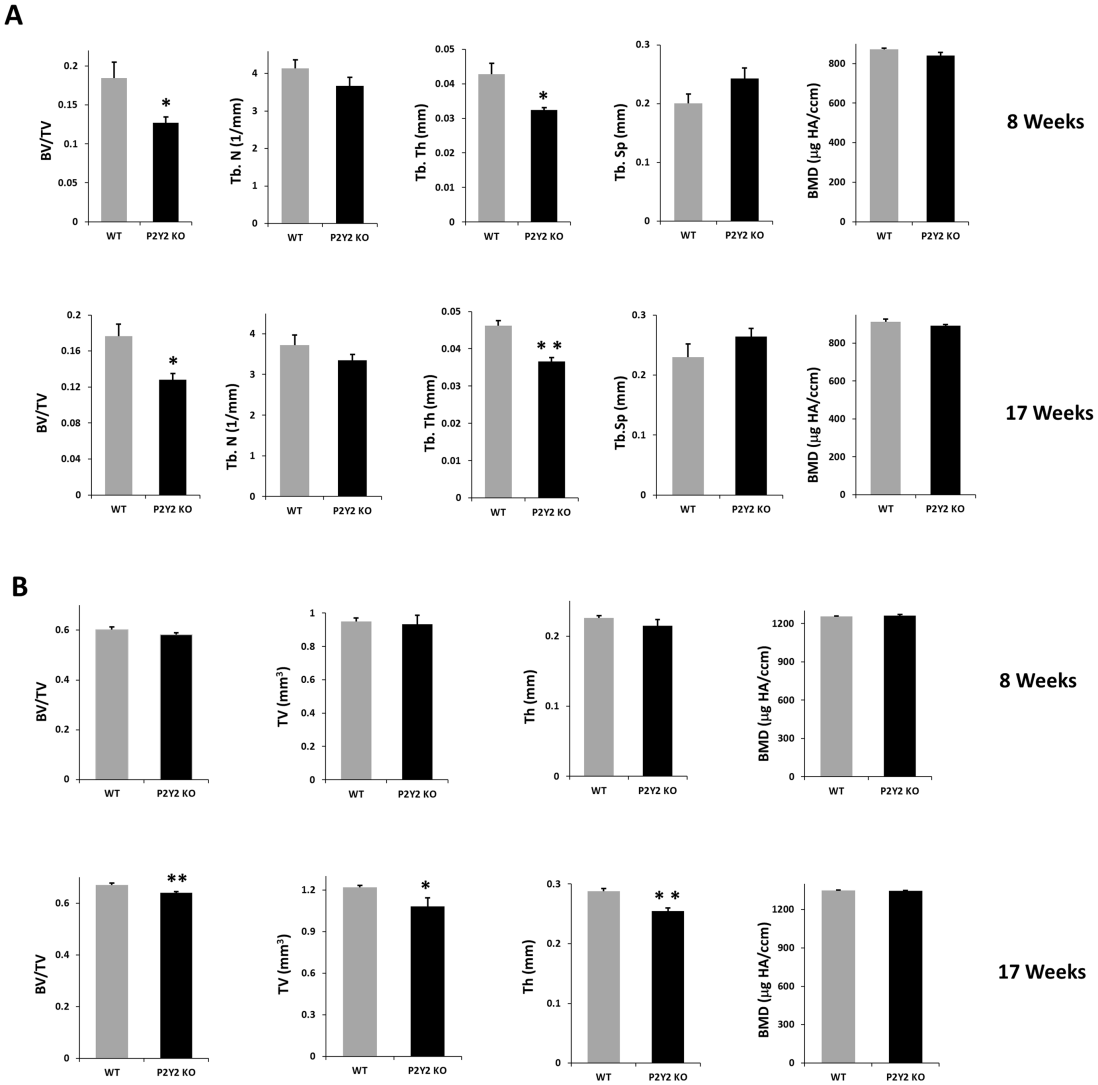
Bone loss in P2Y_2_ KO age-matched WT and P2Y_2_ KO male mice (8 and 17 weeks of age) were subjected to micro-CT analysis. (A) Parameters of trabecular bone mass, including bone volume fraction (BV/TV), trabecular number (Tb. N), trabecular thickness (Tb. Th), trabecular separation (Tb. Sp), and bone mineral density (BMD) were quantified; (B) Parameters of cortical bone quantified included bone volume fraction (BV/TV), total volume (TV), cortical bone thickness (Th), and bone mineral density (BMD). (n = 4–6, *p<0.05, **p<0.01) Error bars represent SEM.

To examine the bone phenotype of P2Y_2_ KO mice, we used micro-CT to analyze femoral structure of WT and P2Y_2_ KO mice at age of 8 and 17 weeks. We found that trabecular bone volume and thickness are significantly decreased in P2Y_2_ KO mice relative to WT mice at both 8 and 17 weeks of age (Fig 1A). For cortical bone, there were no significant differences between WT and P2Y_2_ KO mice at 8 weeks. However, at 17 weeks, the cortical bone volume and mean cortical thickness are significantly decreased in P2Y_2_ KO mice (Fig 1B).

### Three-Point Bending Test of Bone Mechanical Properties

**Figure 2:**
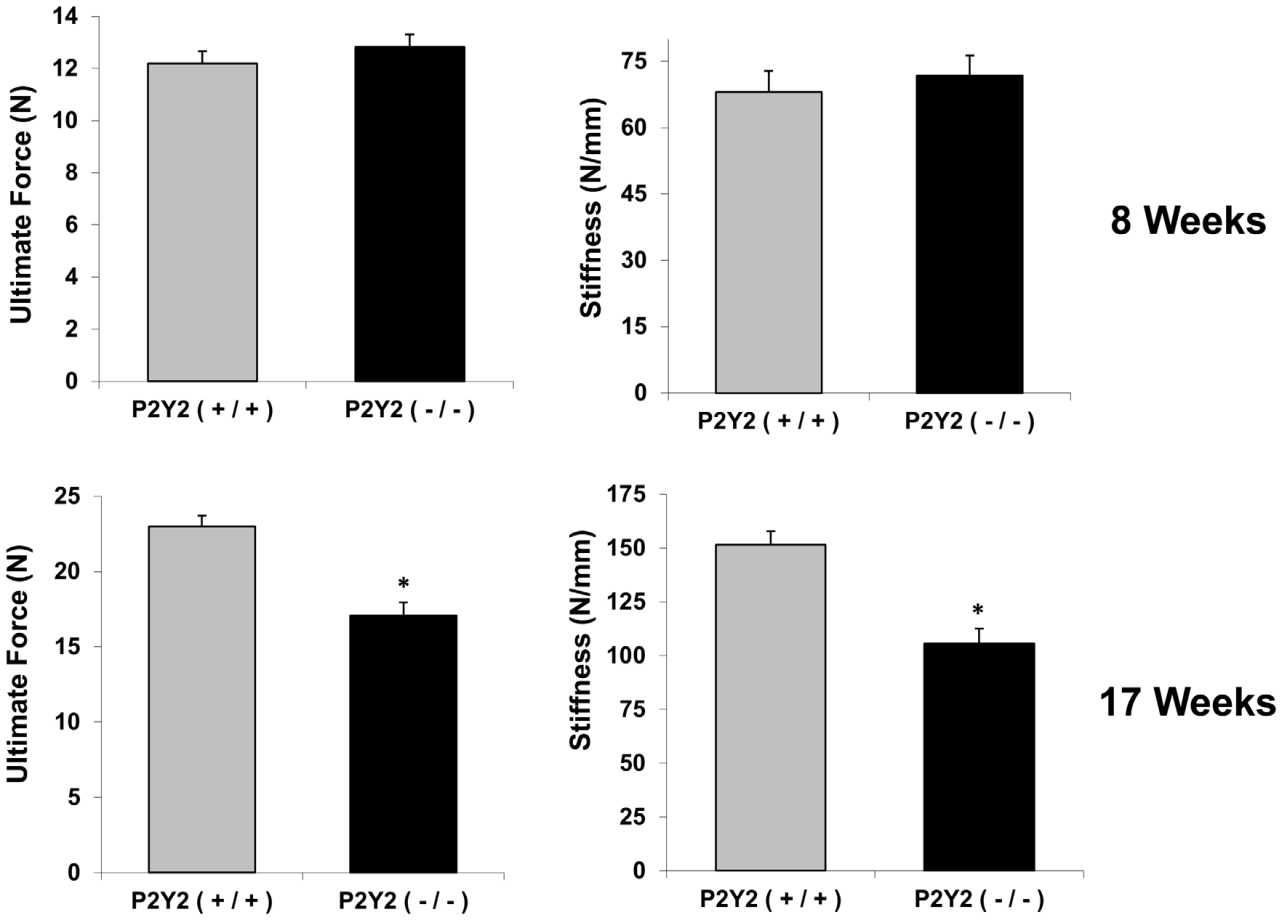
Mechanical properties of cortical bone were decreased in P2Y_2_ KO mice. Femurs of age-matched WT and P2Y_2_ KO male mice (8 and 17 weeks of age) were subjected to three point bending. Ultimate force and stiffness were recorded. (n = 6–8, *p<0.05) Error bars represent SEM.

To further confirm bone structure difference between WT and P2Y_2_ KO mice, we employed three-point bending experiments to measure the mechanical properties of mouse femurs. At 8 weeks the ultimate force and bone stiffness were not different between bones from WT and P2Y_2_ KO mice. However, at 17 weeks, P2Y_2_ KO mice showed significantly lower bone ultimate force and stiffness than WT mice (Fig 2).

### Alkaline phosphatase, calcium staining and osteocalcin mRNA of bone marrow cells from WT and P2Y_2_ KO mice

**Figure 3:**
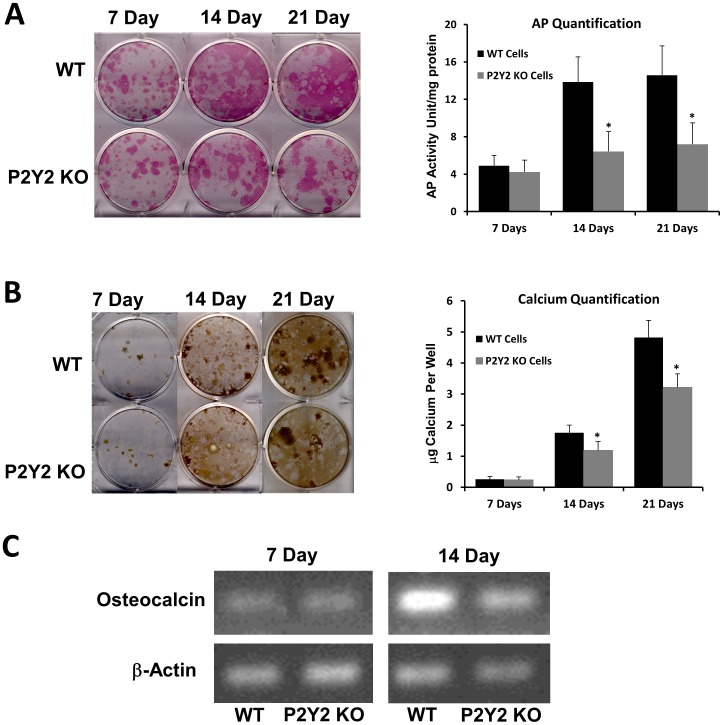
Differentiation (A, C) and mineralization (B) of bone marrow cells was decreased in P2Y_2_ KO mice. (A) Left: Images of AP staining from bone marrow cell cultures; Right: Bar graph representation of AP quantified by colorimetric conversion of p-nitrophenol phosphate to p-nitrophenol and normalized to total protein. (B). Left: Images of von Kossa staining from bone marrow cell cultures; Right: Bar graph representation of calcium qualification by o-cresolphthalein method in each well. (C). Represented images of 35 cycles of RT-PRC show the osteocalcin mRNA levels in bone marrow cells from both WT and P2Y_2_ KO mice (β-actin as controls). (n = 4, *p<0.05) Error bars represent SEM.

The same amount of bone marrow cells from WT and P2Y_2_ KO mice were cultured in 6-well plates for 7, 14 and 21 days in cell differentiation media. Subsequently, cells were stained for AP. There was no difference in AP activity at day 7 between WT and P2Y_2_ KO cells. However, the AP activity in WT cells was significantly higher than that in P2Y_2_ KO mice at days 14 and 21 (Fig 3A). Similarly, we used von Kossa staining to examine calcium deposition during marrow cell differentiation. Cells from WT mice deposited significantly more calcium at 14 and 21 days than did cells from P2Y_2_ KO mice (Fig 3B). Additionally, we examined another osteoblast-related gene osteocalcin changes and found that there was no difference in the mRNA levels of osteocalcin at day 7 between WT and P2Y_2_ KO cells. However, the osteocalcin mRNA levels in WT cells were significantly higher than those in P2Y_2_ KO cells at day 14 (Fig 3C). The results suggest that bone marrow cells from P2Y_2_ KO mice, relative to those form WT mice, display a decreased ability to differentiate into osteoblastic cells. This may at least partially explain their osteopenic phenotype.

When measuring AP activities, we also determined total protein contents in each well. We found the total proteins from wells of WT cells were significantly higher than those from P2Y_2_ KO cells at 7 days (345±41 µg/well vs. 272±24 µg/well) and 14 days (838±64 µg/well vs. 617±49 µg/well), respectively, suggesting proliferation deceases in P2Y_2_ KO cells.

### Response of primary osteoblastic cells ATP and mechanical stimulation

**Figure 4:**
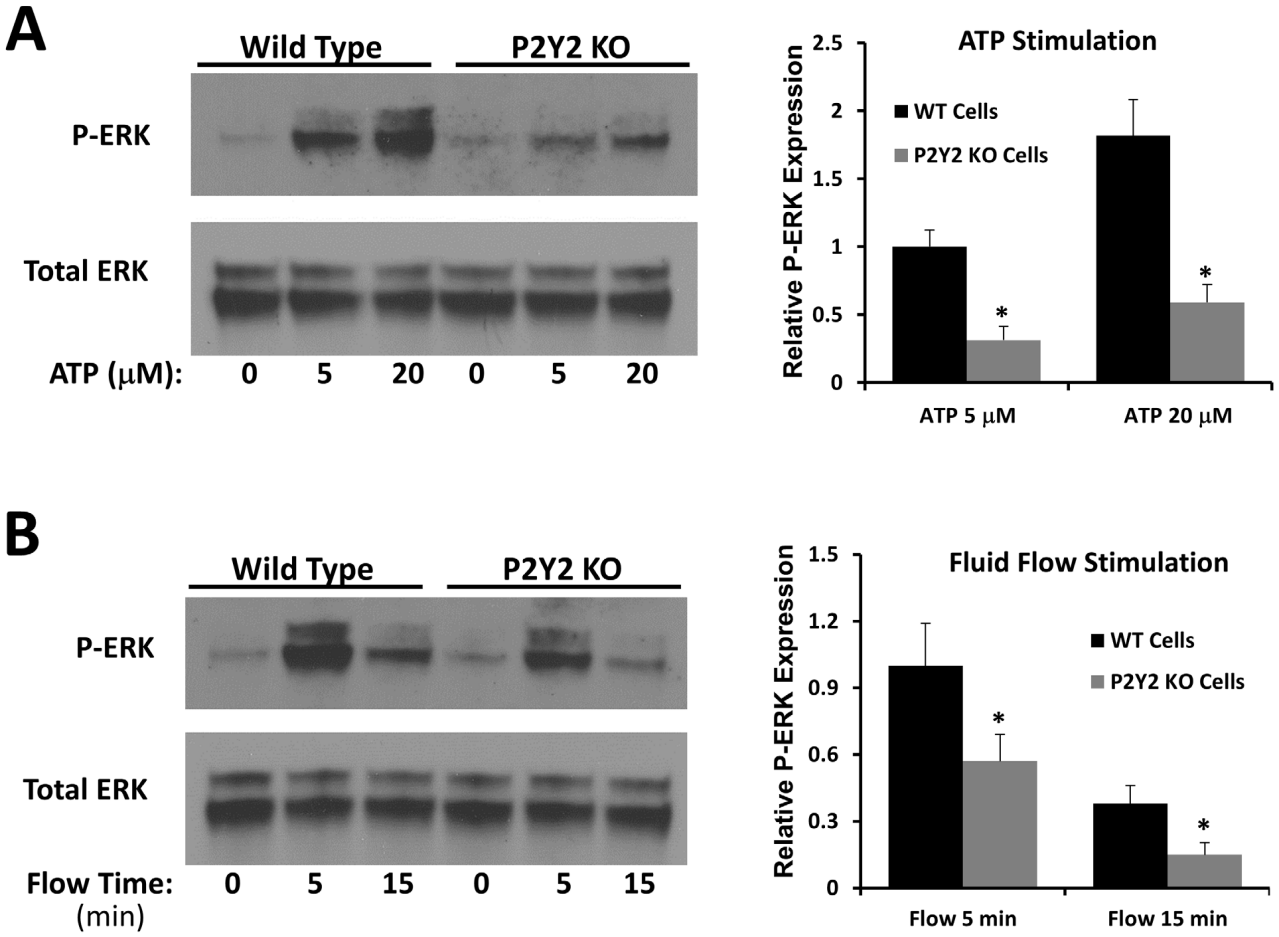
Response to ATP (A) and oscillatory fluid flow (B) was decreased in P2Y_2_ KO mice. (Left: western blot analysis of ERK1/2 phosphorylation in primary osteoblastic cells. Right: Bar graph representation of ERK1/2 phosphorylation quantified by scanning densitometry normalized to total ERK1/2). (n = 4–6, *p<0.05) Each bar represents SEM.

To examine the mechanism underlying the osteopenic phenotype of P2Y_2_ KO mice, we examined the response of osteoblastic cells from these mice to both ATP and mechanical stimulation. Osteoblastic cells from WT and P2Y_2_ KO mice were subcultured into 6-well plates. After 3 days, we added ATP at concentrations of 5 µM or 20 µM directly to plates containing osteoblastic cells from WT and P2Y_2_ KO mice. ATP stimulated ERK1/2 phosphorylation was significantly greater in WT cells relative to P2Y_2_ KO cells (Fig 4A).

Additionally osteoblasts from WT and P2Y_2_ KO mice were subcultured on glass slides as described before. After 3 days, the slides were exposed to oscillatory fluid flow at 10 dynes/cm^2^ for 5 minutes. Western blot analysis revealed that fluid flow stimulated ERK1/2 phosphorylation was significantly greater in WT cells relative to P2Y_2_ KO cells (Fig 4B). The results suggest that osteoblastic cells from P2Y_2_ KO mice are less sensitive to mechanical stimulation, which may at least partially explain their bone phenotype.

### ATP releases in response to mechanical stimulation

To further investigate role of P2Y_2_ in bone mechanotransduction, we examined ATP releases from osteoblastic cells from WT and P2Y_2_ KO mice exposed to fluid flow. ATP release from WT and P2Y_2_ KO cells was not statistically different at the first minute when the highest ATP concentration was measured (12.65±3.16 nM and 11.97±3.47 nM, respectively).

## Discussion

Extensive studies have sought to discover the mechanotransduction pathways in bone and bone cells, but the exact underlying mechanisms are still elusive. To date, researchers have focused on the roles of integrin, membrane channels, primary cilium etc to study bone mechanobiology [12]–[14]. Given the complexity of mechanical transduction, it is no surprise that several pathways are involved in this process. It is well known that mechanical stimulation is able to induce the release of ATP molecules from bone cells, which subsequently activate P2 purinergic receptors [26]. Accumulating evidence suggests these P2 purinergic receptors play an important role in bone cell mechanotransduction [15], [17].

We have previously shown that P2Y_2_ purinergic receptors are responsible for oscillatory fluid flow induced intracellular calcium mobilization in MC3T3-E1 osteoblast cells [4], [5]. P2Y_2_ receptors also have an important role in regulating ERK1/2 phosphorylation in bone and chondrocyte cell lines in response to mechanical stimulation [27], [28]. In this study, we showed that osteoblastic cells from P2Y_2_ KO mice have a weaker response to ATP stimulation than those from WT mice. This result was not surprising as ATP is able to initiate ERK1/2 phosphorylation and P2Y_2_ receptors are one of the major ATP receptors in osteoblasts. Previously, researchers have demonstrated that flow induced ATP release in endothelial cells and smooth muscle cells is able to act on P2 receptors to initiate cellular responses which ultimately regulate vascular tone and blood flow [18], [19]. In addition, it has been reported that ATP released by chondrocytes in response to mechanical loading or inflammation contributes to cartilage tissue destruction by activating signaling pathways involved in articular pathology [20]. These pieces of evidence suggested the importance of P2 receptors in mechanotransduction of different cell types.

Our studies suggest that bone marrow stromal cells from P2Y_2_ KO mice display decreased proliferation and osteogenic differentiation rate relative to those from WT mice. The data may explain the importance of ATP release in response to fluid flow for bone marrow stromal cell proliferation as shown in a previous study [29]. According to some earlier studies, P2Y_2_ receptors play an important role in differentiation of several other cell types, including neurons [30], [31]. P2Y_2_ may also be involved in bone marrow cell differentiation into osteoblasts, although there is a possibility that the strong differentiation ability in WT cells is enhanced by high cell density due to their higher proliferation rate.

Our *in vitro* results of differentiation and mineralization were in contrast to what Orriss et al. have previously reported [32]. This may be due to the different cells used in their study, in which rat primary calvarial osteoblasts were treated with ATP or UTP, suggesting the role of P2Y_2_ receptors in mineralized bone formation. It is well known that ATP and UTP can activate different subtypes of P2X and P2Y receptors, thus the responses of ATP and UTP may not accurately reflect the role of only P2Y_2_ receptors. However, our approach was to employ P2Y_2_ deficient bone marrow stromal cells to elucidate the role of P2Y_2_ receptors in bone mineralization. Moreover, heterogeneous cell populations of bone marrow stromal cells were more likely to mimic *in vivo* environment for studying bone mineralization.

In addition to cell based studies *in vitro*, we also investigated the difference in bone structure between WT and P2Y_2_ KO mice by micro-CT analysis. We found there are no significant differences between these mice in terms of cortical bone total bone volume and mean cortical bone thickness when the mice were 8 weeks old. But interestingly, cortical bone from 17 week old P2Y_2_ KO mice displayed significantly less total bone volume and bone thickness than did WT mice. The results suggest that the result of P2Y_2_ receptor deficiency does not manifest until a more advanced age. This may be because it takes some time for the decreased responsiveness to mechanical load to affect bone phenotype. Interestingly, we did not found any significant difference in BMD between wild type and P2Y_2_ KO mice, which conflicts with previous report [33]. This may be due to different genetic background of the inbred strains used. However, our results of reduced bone volume in P2Y_2_ KO mice are consistent with other in vivo studied in P2Y_1_ and P2X_7_ [21], [34], other subtype P2 receptors. More importantly, both P2Y_2_ KO mice were P2Y_2_ deficient in all cell types. Any environment or other tissue changes may complicate experimental results in bone. Future mouse model study of P2Y_2_ deficient only in osteoblast/osteocytes will elucidate the roles of P2Y_2_ receptor in bone biology.

We further examined ultimate force and stiffness of bones, through 3-point bending experiments, from these two types of mice. Our results showed that there is no significant difference in either ultimate force or stiffness between WT and P2Y_2_ KO mice at 8 weeks, which is consistent with our micro-CT data (no significant differences in cortical parameters between WT and KO mice at 8 weeks). But at 17 weeks, both ultimate force and stiffness of femurs from P2Y_2_ KO mice were dramatically decreased compared to those in WT mice. This may have resulted from the loss of bone volume and bone thickness in 17 week old P2Y_2_ KO mice as revealed by micro-CT. The difference between 8 and 17 weeks of cortical bones is that it takes some time for the decreased responsiveness to mechanical load to affect bone phenotype. To fully understand the molecular mechanisms of P2Y_2_ receptor in bone mechanobiology, our ongoing and future approaches will examine *in vivo* bone formation rates at 8 and 17 weeks, and investigate mechanical loading induced bone formation at 17 weeks. Due to the fact that it takes a long time to observe a difference in bone structure changes between WT and KO mice, unlike *in vitro* cell models we may not see a difference in bone formation changes *in vivo* by using dynamic histomorphometric measurements in a few days. Thus, ideal approaches will be to examine *in vivo* bone formation under mechanical challenging (mechanical loading) in mice which are P2Y_2_ deficient only in osteoblast/osteocytes eliminating other tissue effects.

ATP is a well-known local mediator in bone and can initiate a series of other cellular activities, such as intracellular calcium mobilization and ERK1/2 phosphorylation as shown in our previous studies [4], [5]. ATP works through P2 receptors, which are divided into two subclasses, P2X and P2Y. Earlier studies have demonstrated that P2X_7_ is important for maintaining bone sensitivity to mechanical stimulation through PGE2 signaling pathways [21] and P2Y_1_ is able to enhance bone resorption through the RANKL pathway [35]. In addition, our results suggest that P2Y_2_ receptors are one of the major contributors to osteoblast mechanotransduction through ERK1/2 pathways. Our AP, osteocalcin and total protein results suggest the absence of P2Y_2_ receptors in bone tissue results in decreased sensitivity to loading as well as slower bone cell differentiation and proliferation.

We found that fluid flow-induced ATP release from WT or P2Y_2_ KO osteoblastic cells was the same, strongly suggesting that the effects observed in this study are directly due to the lack of P2Y_2_ receptor, instead of ATP release. Our previous studies suggest that membrane structural components related to lipid rafts, cholesterol and GPI-anchored proteins may play an important role in mechanically induced ATP releases in osteoblastic cells [27]. Thus, our results suggest that P2Y_2_ receptors may not be involved in mechanically induced ATP releases, but involved in subsequent responses after released ATP stimulation.

In conclusion, we demonstrated a reduction in differentiation and mineralization in bone marrow cells and decrease in response to ATP and fluid flow in primary osteoblastic cells from P2Y_2_ KO mice, relative to those from WT mice. These changes may account for the bone phenotype observed in 17-week old P2Y_2_ KO mice and our *in vitro* results may imply an important role of P2Y_2_ in bone mechanotransduction.
